# The Role of Segmented Filamentous Bacteria in Immune Barrier Maturation of the Small Intestine at Weaning

**DOI:** 10.3389/fnut.2021.759137

**Published:** 2021-11-18

**Authors:** Linda A. Oemcke, Rachel C. Anderson, Eric Altermann, Nicole C. Roy, Warren C. McNabb

**Affiliations:** ^1^Riddet Institute, Massey University, Palmerston North, New Zealand; ^2^School of Food and Advanced Technology, Massey University, Palmerston North, New Zealand; ^3^Smart Foods Innovation Centre of Excellence, AgResearch, Palmerston North, New Zealand; ^4^Consumer Interface Innovation Centre of Excellence, AgResearch, Palmerston North, New Zealand; ^5^High-Value Nutrition National Science Challenge, Auckland, New Zealand; ^6^Department of Human Nutrition, University of Otago, Dunedin, New Zealand

**Keywords:** gastrointestinal tract, segmented filamentous bacteria, interleukin 17, immunoglobulin A, immunological barrier, food substrate, weaning diet

## Abstract

The microbiological, physical, chemical, and immunological barriers of the gastrointestinal tract (GIT) begin developing *in utero* and finish maturing postnatally. Maturation of these barriers is essential for the proper functioning of the GIT. Maturation, particularly of the immunological barrier, involves stimulation by bacteria. Segmented filamentous bacteria (SFB) which are anaerobic, spore-forming commensals have been linked to immune activation. The presence and changes in SFB abundance have been positively correlated to immune markers (cytokines and immunoglobulins) in the rat ileum and stool samples, pre- and post-weaning. The abundance of SFB in infant stool increases from 6 months, peaks around 12 months and plateaus 25 months post-weaning. Changes in SFB abundance at these times correlate positively and negatively with the production of interleukin 17 (IL 17) and immunoglobulin A (IgA), respectively, indicating involvement in immune function and maturation. Additionally, the peak in SFB abundance when a human milk diet was complemented by solid foods hints at a diet effect. SFB genome analysis revealed enzymes involved in metabolic pathways for survival, growth and development, host mucosal attachment and substrate acquisition. This narrative review discusses the current knowledge of SFB and their suggested effects on the small intestine immune system. Referencing the published genomes of rat and mouse SFB, the use of food substrates to modulate SFB abundance is proposed while considering their effects on other microbes. Changes in the immune response caused by the interaction of food substrate with SFB may provide insight into their role in infant immunological barrier maturation.

## Introduction

It is widely accepted that, amongst other factors, the microbial consortia in all regions of the body contribute to maintaining homeostasis and good health (1–4). These positive effects, specifically of the gastrointestinal tract (GIT) microbes, created an interest in how the microbiota interacts with host cells. Most of the knowledge regarding upper and lower GIT microbiomes comes from research using fecal samples, and the knowledge of upper GIT communities comes from research using samples collected from those sites (5–7). Feces have been used to represent microbes of the lower GIT. Some studies have also used feces as a proxy of the upper GIT (8, 9) as the human small intestine is more difficult to access even with the use of invasive techniques (10, 11). Nevertheless, some microbes present in high abundance in the small intestine have caught scientists' attention, as emerging evidence in their involvement with the immune system points to their significance (12–16).

Segmented filamentous bacteria (SFB) were discovered attaching to the ileal mucosa in invertebrates (17) and healthy vertebrates (12–16, 18). These Gram-positive, anaerobic, spore-forming, commensal microbes have been classified within the Firmicutes phylum and are within the *Clostridiaceae* family. SFB possess a unique holdfast structure which facilitates their attachment to the ileal mucosa. During the lifecycle of SFB, the segmented filaments divide and elongate, forming viable intracellular offspring or spores in adverse conditions (15). The classification of SFB sparked an interest in their function as they were seen to occupy the distal ileum (19, 20). In this region, SFB attach to the ileal epithelium overlying the Peyer's patches where naïve T cells undergo antigen-driven activation and expansion to yield T helper cells (14, 19). Studies reported a positive correlation of SFB abundance with the concentration of interleukin 17 (IL-17) in plasma (20–24) and a negative correlation of immunoglobulin A (IgA) in feces and ileum contents of 4–6 week old mice (25–27), though this is not exclusive to SFB (5, 28–30). Interestingly, these effects occur without signs of systemic inflammation in the host (20–24).

**Figure 1:**
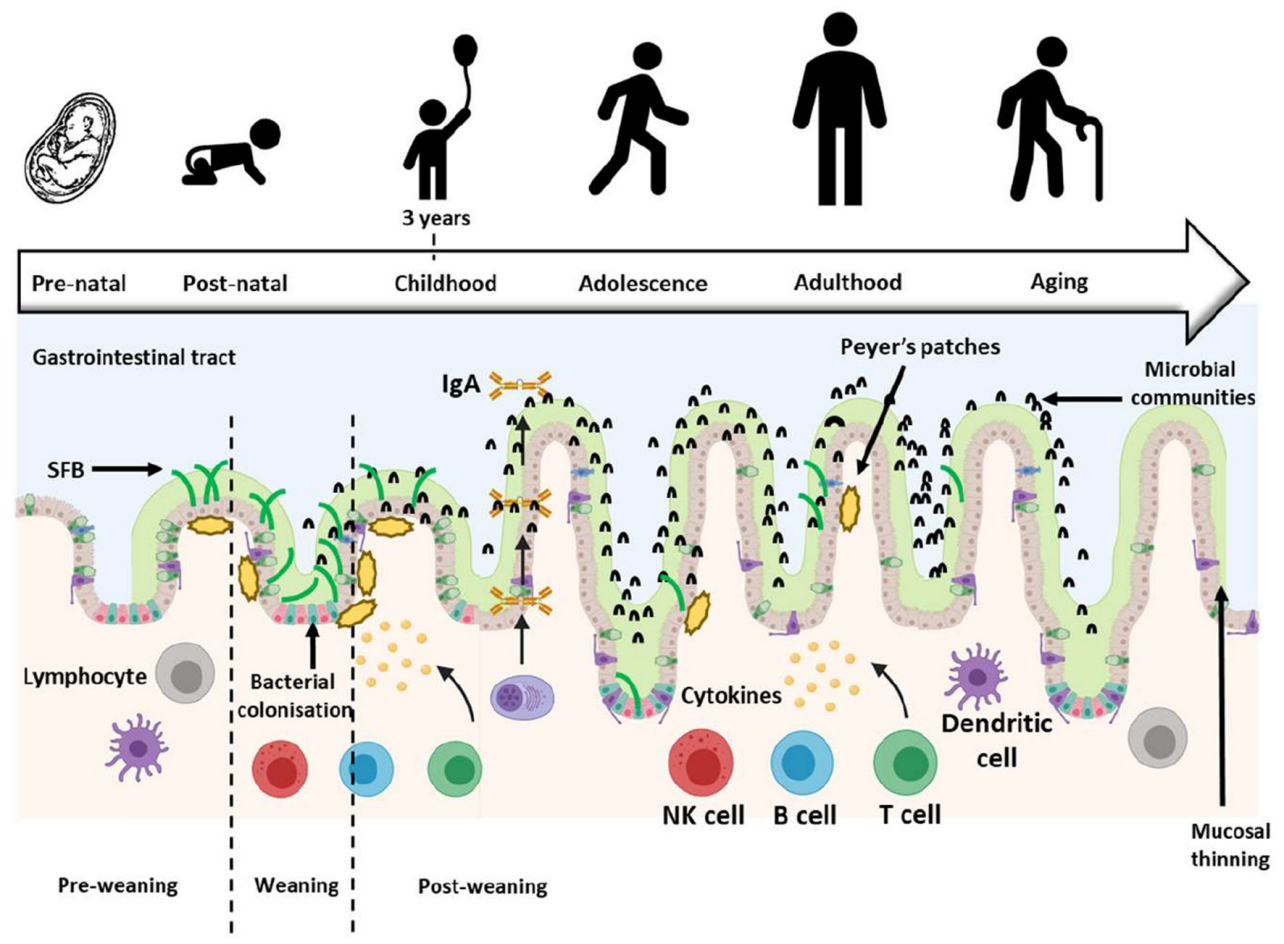
Development of the gastrointestinal tract (GIT) throughout life. The GIT barriers continue developing and maturing postnatally until about 3 years of age when the resident microbiota resembles that of an adult. During this time, SFB abundance changes, first increasing and then decreasing at weaning, but SFB abundance remains low into adulthood [Figure adapted from Jašarević et al. (35); Created with BioRender.com and PowerPoint]. SFB, Segmented filamentous bacteria; IgA, Immunoglobulin A.

Additionally, the presence of SFB is variable; their abundance in the ileum of infants increases at weaning from 6 months, peaks around 12 months and plateaus until 25 months post-weaning (24, 31, 32). A similar pattern has been reported in BALB/c mice (25), ICR (Institute of Cancer Research) mice (32) and Sprague-Dawley rats (18) where SFB abundance increased at weaning from 20 days, peaked around 24–28 days and plateaued until 50 days post-weaning. This timing of abundance change corresponds with the infant transitioning from a milk-based diet to one which increasingly includes solid foods. The increased dietary complexity is known to contribute to the immune system maturation progression and drives colonization by a different and more diverse microbiota in the GIT (31, 33, 34) possibly including SFB. Figure 1 details the abundance of SFB as suggested to be involved in infant GIT immune maturation.

The reports of positive or negative correlation of IL-17 and IgA production with abundance changes of SFB in ileal contents or stools at weaning created an interest in studying these microbes and their influence on the infant GIT immunological barrier development. *In vitro* studies were carried out initially and proved challenging as SFB did not grow outside the ileum (36, 37). SFB are reportedly anaerobic yet attach to epithelial cells that require oxygen. The attempts to further investigate the effects of SFB on the immune system *in vitro* became a significant technical challenge due to the poor viability of these microbes. SFB were therefore studied using animal models (18, 25, 36, 37) and stool samples from human participants (10, 32, 38).

Ohashi et al. (25) investigated IgA concentration and SFB abundance from ileal contents in weanling mice. Their study reported that SFB abundance exhibited a temporal profile; it increased post-weaning immediately, peaked, then decreased and plateaued. Additionally, the concentration of IgA showed a negative correlation with SFB abundance. A study also reported that the abundance of SFB was higher in the ileum of 4 and 8-week-old mice fed a composite diet based on natural ingredients when compared to mice fed a solely milk powder diet. However, when purified diets containing the macronutrients from the composite diet were tested, no fat, carbohydrate or fiber fraction appeared to influence SFB abundance. Whilst it was not possible to identify a single nutritional factor responsible for SFB abundance, it was clear that composition of complex diets was at least one factor responsible for SFB abundance (39). Consideration should be given to the potential of various food substrates to increase the abundance of ileal SFB and related changes in markers of immune maturation during the weaning transition.

Further to that, analysis of the SFB genome revealed enzymes involved in metabolic pathways which these microbes utilize for survival, growth, and development (4, 40, 41). Data sets of enzymes in the glycolytic and pentose phosphate pathways of SFB predict the use of some of the by-products in metabolic pathways for synthesizing amino acids, vitamins, and cofactors within the genome (40, 41). The genome of SFB is smaller (~1.57 Mb) compared to that of their relatives *Clostridium* (3.97 Mb). The genome reportedly lacks genes for biosynthesis of most amino acids. Amino acid transporters and permeases found in the genome imply SFB uptake amino acids possibly from dietary sources and host protein degradation by proteases secreted by SFB. The absence of these important genes could explain their commensal nature (40) and possibly reflect the size of the genome. Attachment of SFB to the ileal mucosa and abundance change following the introduction of solid foods suggest the genome may not support all functions required to be free-living. However, genome size does not determine whether a microbe can be free-living or not.

This narrative review analyzes the suggested role of SFB in infant immunological barrier maturation and whether modulation of SFB abundance could positively impact immune maturation of the small intestine for later health benefits. Referencing the published genomes of rodent SFB, the use of food substrates to potentially modulate SFB abundance is proposed while also considering the effects on the immune system and other GIT microbes.

## Development and Maturation of the Small Intestinal Barrier

The small intestinal barrier comprises the microbiological and chemical barriers, as well as the physical and immunological barriers in the mucosa. The microbiological barrier, located above the mucus layers, houses most of the microbes. Some microbes produce antimicrobial peptides that inhibit pathogen attachment by limiting their growth while other microbes are involved in nutrient acquisition and energy regulation (42–45). Below the microbiological barrier lies the chemical barrier that consists of an outer, less viscous mucus layer followed by the inner mucus layer containing fewer microbes. The inner mucus layer contains free-floating microbes and those, such as SFB, which attach to the epithelium.

Below the chemical barrier is the physical barrier known as the epithelium. It comprises columnar epithelial cells organized into crypts and villi (46). At the base of the epithelial crypts, Paneth cells secrete antimicrobial peptides, including defensins, lysozyme and phospholipase, which prevent the growth of pathogenic microbes (47). In the physical barrier, protein complexes provide structural integrity and act as channels that allow or prevent the passage of substances contributing to this barrier's selective permeability (48). The immunological barrier is the innermost layer where the immune system provides defense against pathogens and antigens and exists in an immune-suppressed state maintaining homeostasis even with dietary antigens and microbiota that pass through the physical barrier (49).

Of interest is the immunological barrier where immune cells begin appearing at ~6 months of gestation. Recruitment of epithelial lymphocytes in the GIT begins signaling the functioning of an immature immunological barrier (50). Naïve T cells undergo activation and expansion in the Peyer's patches yielding T helper cells which are later stimulated to produce cytokines involved in immune responses (19). Naïve B cells also begin differentiating into plasma cells that produce immunoglobulins which recognize and bind to pathogenic bacterial or viral antigens and assist in their destruction (51). Fully developed during gestation, the immune system matures post-birth possibly along with the microbiological barrier. Maturation of the immunological barrier is thought to occur at weaning (about 6 months of age) when foods containing antigens and microbes are introduced and may trigger immune responses in the infant GIT. It is during complementary feeding, when solid foods are introduced into the infant's milk-only diet, that the abundance of SFB reportedly increases, peaks, then plateaus. The studies which reported a positive and negative correlation of SFB abundance with IL-17 (20, 21, 52, 53). SFB also reportedly initially induce the production of IgA suggesting a positive correlation (54). However, the continued increase in luminal IgA concentration reportedly results in decreased SFB abundance in a somewhat self-regulating system whereby IgA restrains and possibly prevents an overgrowth of SFB in the ileum. This implies a negative correlation of IgA concentration with SFB abundance. These reported correlations led to the suggestion that SFB may play a role in influencing GIT immune system maturation.

The timing of SFB abundance changes around weaning (18, 25, 32) suggests that the inclusion of complementary foods might be a way to alter their abundance and hence effects on the epithelial and immune cells in the ileum. SFB are proposed to obtain nutrients from the ileal lumen (12, 14, 39, 55–57), and directly from the host as they cannot successfully survive outside the environment of the ileum (36). SFB may have evolved to attach to the ileal mucosa and during complementary feeding potentially derive nutrients in a cross-feeding manner with other bacteria residing in the inner mucus layer of the chemical barrier. If this is the case, then substrates from the diet might modulate SFB abundance at weaning, which had not previously been investigated. Therefore, an approach would be to provide substrates to enhance their abundance and perhaps functionality though only at weaning and immediately post-weaning to avoid any risks posed by a sustained increase in the abundance of SFB.

## Background on Segmented Filamentous Bacteria

**Figure 2:**
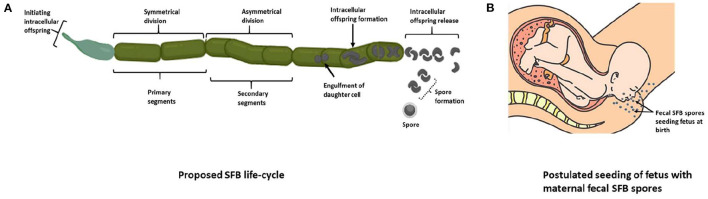
The proposed life cycle of SFB. **(A)** The “holdfast” structure on vegetative segments allows SFB to anchor onto host epithelial cells and lengthen, forming primary and secondary segments (65). These segments double in length and form filaments in which a mother cell forms, engulfing the subsequent daughter cell, which then divides to form two viable intracellular offspring (66). The offspring then exit the filament and later attach to the host epithelium or form spores under stressful conditions (67–69), which may then be transferred to another host of the same species (70). **(B)** SFB fecal spores may seed the oral cavity during vaginal birth, or SFB spores could be passed from the mother to the embryo via the placenta in infants born *via* Cesarean-section. These theories may explain the origin of SFB in infants, though they have not been demonstrated experimentally. The relative abundance of genes predicted to encode cell cycle control functions, envelope biogenesis, and trafficking was higher in the mouse SFB genome than in other clostridia. They may reflect the complex cell differentiation processes during this life cycle (71). Four predicted N-acetylmuramoyl-L-alanine amidases unique to SFB (cell wall hydrolases, PF01510, PF05105, and PF01520) are also hypothesized as necessary in forming the different cell morphotypes and may be responsible for releasing “holdfasts” and spores from the filaments (72) [**(A)** adapted from Schnupf et al. (36); Created with BioRender.com] [**(B)** with permission from EPI-NO. Source: https://www.epino.de/en/birth-preparation.html]. SFB, Segmented filamentous bacteria.

Reported initially over 150 years ago, SFB were observed in the ileum of invertebrates (17), firmly attaching to the epithelial lining (58). SFB were initially given the provisional name *Candidatus Arthromitus* (59). However, the first filamentous bacteria isolated from arthropods, though morphologically similar to those isolated from vertebrates, were shown to belong to the *Lachnospiraceae*, a family within the order C*lostridiales*, after analysis of 16S rRNA gene sequences (58). *Arthromitus* showed an apparent absence of SFB-like 16S rRNA gene sequences present in vertebrate SFB. This suggested different strains of SFB inhabiting invertebrates and vertebrates. Therefore, another taxonomic classification was proposed and accepted (60) to name the species isolated from vertebrates. It was named *Candidatus Savagella* (61) and provisionally classified under Savagellaceae, a credit to Dwayne C. Savage, the American gut microbiologist who first observed and described them in the ileum of rodents (62). Rods and filaments of SFB were identified by light microscopy and fluorescent *in situ* hybridization (32, 63) and later PCR methods were used to detect SFB in the ileum of rats (64). SFB are reported to replicate in the ileum through a life cycle deduced by electron microscopy in rodents (Figure 2A) (73). SFB exist in two forms, a vegetative segment containing a holdfast structure that allows them to anchor to the host epithelial cells, and as spores which are intracellular offspring encapsulated during adverse conditions. These morphologically distinct intrasegmental bodies indicate that SFB exist in vegetative and dormant states (67). Once the SFB intracellular offspring are released, they are transferred to another host of the same species (70). This observation was confirmed by an *in vitro* investigation in SFB gene diversity and host-specificity of four flagellin genes in mice and rats which revealed two relatively conserved and two-variable genes and confirmed the preferential attachment of SFB to the epithelial mucosa of their host (70).

The origin of SFB in the GIT is thought to be via vertical transmission, as with other GIT microbes, from the mother to the fetus. The presence of SFB in dormant stages suggests that their spores found in maternal feces may seed the infant during vaginal birth (Figure 2B). In the case of infants born *via* Cesarean-section, it is unclear how SFB are present in the infant GIT. SFB spores could also be passed from the mother to the embryo via the placenta and may be the origin of SFB in infants born *via* Cesarean-section if SFB are even present in these infants. This theory, however, has no reported evidence.

In postnatal life, SFB are present in the ileum of healthy weanling rodents (25). They have also been detected in infant, and adult humans' feces though collecting and analyzing ileal samples are preferred (32). However, collecting ileal samples in conscious subjects requires invasive diagnostic tools such as scopes (74). Performing these manipulations in infants for routine SFB study is challenging due to the ethical restrictions of researching this age group. Therefore, animal models are currently the most appropriate way to study SFB.

### SFB in Humans

The discovery and characterization of SFB in the ileum of healthy rodents drew interest due to their reported influence on markers of the GIT immune system. Later detection of SFB in the feces of healthy infants and adults suggests that SFB form part of the normal GIT microbiota and, like rodents, may also influence the GIT immune system. Knowledge of the role of SFB in humans is still scarce, and a time-course study elucidating the temporal profile of SFB at the crucial weaning stage in infants has yet to be attempted.

A comparative 16S rRNA gene analysis of SFB in healthy humans feces, mice ilea and chicken ilea indicated similarities in the abundance change of these microbes at similar stages of development (32). The similar findings of SFB abundance in human feces and mice and chicken ilea implied that ileal SFB in humans exhibit a temporal profile of abundance change pre- and post-weaning, and then persist into adulthood and old age as part of the normal microbiota. SFB have also been detected in ileostomy samples of adult patients with ulcerative colitis (64), though the implications of disease-cause by SFB have not been verified. Other than indicating the presence of SFB in the human GIT, these studies provide limited evidence of the interaction of these microbes with the immune system. Current knowledge comes from studies carried out using animal models. However, these observations might not necessarily translate to humans due to differences in the GIT microbiota and immune cell profiles between them.

### SFB and the Immune System

The discovery of SFB in the ileum of vertebrates prompted investigations of their role in the GIT. Studies on how SFB interact with the immune system indicate that SFB do not appear to cause ill-effects in healthy vertebrates.

#### Immunoglobulin A and Interleukin 17

Studies on SFB have investigated their relationship with IgA, the most abundant immunoglobulin occurring in the body. IgA acts to block excessive bacterial adherence or translocation, mediates the neutralization of toxins and viruses, and removes unwanted macromolecular structures on the epithelium in the GIT (75). IgA in infancy reportedly originates from breast milk contributing to high levels found in the GIT lumen during the 1st month which gradually decrease until 5 months of age (76), then remain relatively low and stable until 24 months of age (77). Postnatal microbial colonization and maturation of the GIT stimulate host production of IgA, and the luminal levels slowly increase (78).

The presence of IgA in the GIT is important, especially in infants below 6 months of age with an immature immune system. Interactions between SFB and IgA drew attention following reports of SFB inducing IgA production in Swiss (13, 26), BALB/c (26, 79), and C3H/HeN (27) adult mice mono-associated with SFB. These findings suggest that SFB stimulated the germinal centers in the Peyer's patches (22). Further investigation in rodents showed that IgA production increased with increasing SFB abundance and continued increasing as SFB abundance decreased from about 4 weeks postnatally (25–27). A report on the aberrant expansion of mainly SFB and other anaerobes in the absence of hypermutated IgA in adult C57BL/6 mice (54) points at the function of IgA in regulating the bacterial composition of the GIT. The observation from this study suggests that IgA restrains growth of SFB. Therefore, though studies report an initial positive correlation between SFB and IgA, a further increase in IgA concentration results in decreasing SFB abundance, a negative correlation. IgA concentrations increase or decrease is, however, not exclusively linked to SFB. Other commensals, such as Gram-negative *Morganella morganii* (27) and Gram-positive probiotic *Bifidobacteria* (5, 29, 30), induce the production of IgA. B cells which are the origin of IgA may thus be stimulated by microbial colonization, including SFB, resulting in increased IgA levels (27). The reported correlation of IgA with SFB (13, 26, 27, 79) may be evidence of IgA maintaining a homeostatic balance within the microbiota and remains a point of interest in the suggested role of SFB in postnatal immunological barrier maturation.

Many reports on SFB have focused on their ability to stimulate the production of the pro-inflammatory cytokine IL-17 furthering the interest in their influence on GIT immunity (20, 21, 52, 53). IL-17 is essential for host defense against infection by invading pathogens at mucosal surfaces (80, 81). When fecal microbes, without SFB, from Jackson C57BL/6J mice, were introduced into germ-free (GF) mice, Th17 cells were not induced until SFB was added (21). Mice lacking SFB in their microbiota had fewer Th17 cells in the ileum than mice with a normal SFB population. SFB also specifically induced Th17 cells in the small intestinal lamina propria when introduced into GF Swiss-Webster mice (21). The production of Th17 cells demonstrated maturation of the immunological barrier after SFB were introduced into GF mice. Non-colonized control GF mice were also observed to have no Th17 cells, implying an immature immunological barrier (13, 21).

Reports of the influence of SFB on ileal IL-17 production (21, 52, 53), including the reported mechanisms by which SFB achieve this (82, 83), suggest a positive correlation between both. Immunization of adult mice with SFB flagellins (FliC3) resulted in higher upregulation of small intestine epithelial cell factors controlling the differentiation of Th17 (Duox2, Duoxa2, and Nos2) and also promoting the production of IL-17 (83). Further exploration of IL-17 production by SFB in adult mice has revealed that SFB and host ileal epithelial cells communicate by generating endocytic vesicles at the interface of SFB-epithelial cell synapses. The interaction of SFB with the epithelial cells triggers the formation of endocytic vesicles through clathrin-independent and dynamin-dependent endocytosis. These vesicles contain an SFB cell wall-associated protein (P3340), an immunodominant T cell antigen for generating mucosal Th17 cells (82). The vesicles are released into the host epithelial cells, and P3340 induce activation of lamina propria antigen-specific Th17 cells, and subsequently, IL-17 is produced (84). Thus, these observations indicate that SFB flagellins are involved in upregulating ileal epithelial cell genes, which in turn induce IL-17 production.

Like IgA, Th17 cell production is also not exclusive to SFB (21). This was demonstrated when C57BL/6 GF mice mono-associated with SFB induced Th17 cells to a lesser extent than GF mice colonized by SFB and a more complex microbiota (SFB and eight defined commensals) in the small intestine (21, 85). This reported interaction between SFB and other microbes in IL-17 production highlights the synergy among commensals and their influence on the immune system, including Th17 cells. Alternatively, differentiation of Th17 cells was induced in Taconic B6 and Jackson B6 mice treated with antibiotics and then exposed to normal specific-pathogen-free bacteria (52). Upon further investigation, members of the *Bacteroidetes* phylum were reported to be involved indicating that SFB may not be the only microbes capable of inducing IL-17 production.

Overall, the literature reports that SFB-upregulated epithelial cell factors are involved in IL-17 production in adult mice. These observations in adult mice imply the presence of a mature GIT is required and this needs to be considered when investigating the role of SFB in immunological barrier maturation.

#### Immune-Mediated Disease

Research into SFB has highlighted their association with both disease cause (64, 86–88) and protection (89, 90). The findings hint at the abundance of SFB, maintaining a delicate balance between these microbes and the host immune system.

The effort to decipher the role of SFB arose from studies which investigated the involvement of SFB in several functional GIT and autoimmune diseases. Studies with immunodeficient adult mice colonized with only specific-pathogen-free bacteria, only SFB, or a combination of both reportedly developed clinical signs of colitis (87), suggesting that dendritic cells were activated by their colonization of the ileum (91). SFB were also detected in ileal mucosa samples of adult patients with ulcerative colitis. However, their presence might not be linked to the disease (64), and it is hypothesized that the patients' samples might have simply exhibited a higher SFB load (64). It is plausible that higher numbers of SFB induced the production of IL-17 to abnormal levels (86, 92) though this was not measured. A higher abundance of SFB was also reported in fecal samples of adult patients with diarrhea-associated irritable bowel syndrome than those with constipation-associated irritable bowel syndrome (88). Autoimmune diseases have also been associated with SFB where the Th17 cell population in GF adult mouse models of human arthritis and multiple sclerosis inoculated with SFB provoked an onset of the diseases (86, 93). These reports highlight the complexities of the suggested effects of SFB in disease and cannot be restricted to a single type of pathology, nor infer causality.

**Figure 3:**
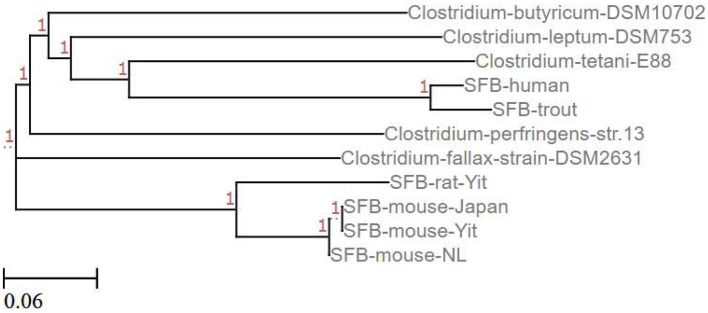
Phylogenetic tree relating SFB to Clostridium species. This phylogenetic tree shows the relationship between complete (SFB mouse-Yit, SFB-rat-Yit, SFB-mouse-Japan, and SFB-mouse-NL) and partial (SFB-human and SFB-trout) SFB genomes and some pathogenic (C. perfringens, C. fallax, and C. tetani) and non-pathogenic (C. butyricum and C. leptum) Clostridium species published in the NCBI database (Created using the EMBL-EMI Multiple Sequence Alignment Clustal Omega tool and ETE toolkit-Phylogenetic Tree viewer).

Mining of sequenced and annotated rat and mouse SFB genomes revealed that SFB lack the genes encoding for known toxins and virulence factors present in pathogenic *Clostridia* (74). This observation and the absence of apparent inflammatory reactions where SFB colonize the ileum suggest that SFB may stimulate IL-17 production without pathological consequences. Phylogenetic analysis of whole SFB genomes indicates that SFB and pathogenic *Clostridia* such as *C. tetani, C. perfringens*, and *C. fallax* share a common ancestor though distantly (Figure 3). Sequencing of SFB isolates from stool samples of healthy adults (32) and those with ulcerative colitis (64) would be required to identify genomic differences between SFB genome-types.

Comparison of genome sequences among SFB filaments isolated from SFB-monocolonized mice along with published SFB genome sequences revealed the presence of single nucleotide polymorphisms (SNPs) (71). SNPs occur frequently and, in some cases, cause missense mutations with no effect, but they can also cause non-sense mutations which affect gene functionality and alter phenotypes. Pamp et al. (71) combined reads, *de novo*, from five individual SFB filaments (SFB-1 to SFB-5) from SFB-monocolonized mice to form the genomic co-assembly “SFB-co.” A second co-assembly, “SFB-mouse-SU,” which was closely related to their SFB was also assembled using the published SFB mouse genome [SFB-mouse-Yit (AP012209)] (40). Some of the loci exhibiting SNPs in the individual SFB filaments included genes encoding for oxaloacetate decarboxylate alpha (OadA), pyruvate kinase (PK) and flagellar motor switch protein (FliN). A conserved lysine residue in OadA, which generates pyruvate from oxaloacetate, was substituted by threonine. A similar mutation in *Vibrio cholerae* renders OadA ineffective. For PK which catalyzes phosphoenolpyruvate to pyruvate, a valine residue was changed to alanine. The SNP in FliN, which together with FliG and FliM forms the switch complex that controls the direction of flagella rotation, resulted in a predicted threonine to alanine change (40). These mutations of FliN in bacteria are reported to result in failure in flagella export and rotation (94, 95). The polymorphisms observed among the five SFB filaments are likely minor variants which coexist within a population of SFB in an animal colony. Multiple genome sequence comparisons of other SFB genomes revealed chromosomal features whereby the highest variability include CRISPR-arrays, phage-related genes and hypothetical proteins (71) which indicate heterogeneity and evolution of SFB lineages within colonies of similar species.

Finotti et al. (64) also sequenced SFB PCR amplicons from colorectal biopsy samples of 35–70 year-old males and females with ulcerative colitis. The sequences were compared to whole-genome sequences of SFB from healthy mouse, rat, turkey, and a partial human SFB sequence in the NCBI Reference Sequence Database (Table 1). Results highlighted nucleotide changes in the ulcerative colitis SFB sequences at positions 64, 68, 81, and 85 reflecting amino acid differences from aromatic to branch-chain, negatively charged to uncharged, aromatic to aliphatic and hydrophobic to positively charged, respectively. The sequenced colorectal SFB genes were representative of SFB from the ileum, but without further information, it is unclear whether ileal and colorectal SFB are genetically similar or not. The lack of complete SFB sequences from healthy humans in the database creates a challenge in making informed comparisons between SFB isolated from healthy vs. diseased adults.

**Table 1:** Nucleotide changes observed in human SFB gene from the terminal ileum of 35–70-year-old males and females with ulcerative colitis (64) compared to codons from the SFB genes of healthy rat and mice.

**Nucleotide position**	**64**	**68**	**81**	**85**
Rat	A**T**C	G**AT**	**A**TA	C**A**T
	Isoleucine (I)	Aspartate (D)	Tyrosine (Y)	Methionine (M)
Mouse	T**T**C	G**A**T	**A**TA	C**C**T
	Phenylalanine (F)	Aspartate (D)	Tyrosine (Y)	Leucine (L)
Human (ulcerative colitis patients)	A**T**T	G**G**A	**G**CC	A**A**A
	Isoleucine (I)	Glycine (G)	Alanine (A)	Lysine (K)

*The nucleotide positions correspond with the nucleotide bases in bold in the amino acid codons. Letters in parentheses are the single-letter abbreviations of the amino acids*.

SFB have been associated with disease protection against type 1 diabetes (89) and rotavirus infection (90) in adult mice. Non-obese diabetic (NOD) adult mice inoculated with SFB were reported to have high levels of IL-17-expressing CD4^+^ cells when compared to SFB-negative NOD mice. The SFB-positive NOD mice also did not develop diabetes though levels of insulitis, a marker for type 1 diabetes, were similar to those in SFB-negative NOD mice. This result implies that SFB colonization may not block the trigger of diabetes but might modulate the progression of the disease (89). Also, the comparison of two different SFB strains administered to GF Rag1-knockout (lack mature B or T cells) adult mice showed reduced rotavirus infectivity. The mechanism by which this effect happened was independent of Th17 cells as SFB administration promoted enterocyte proliferation, migration, and luminal shedding of rotavirus-infected cells. The observations hinted that SFB may prevent infection by hindering rotavirus from utilizing a surface component to bind to the ileal epithelial mucosa. The protective effect against rotavirus infection was, however, conferred more strongly by one of the two SFB strains administered to rotavirus-susceptible Rag1-knockout adult mice, highlighting a potential role of strain-specific phenotypes (90). These reports indicate synergies possibly among SFB strains, along with other microbes, cell signaling receptors and immune system mediators. IgA production was not assessed in the mice as a previous study by Corthesy et al. (96) indicated that IgA contribute to rotavirus protection via intracellular neutralization and not *via* immune exclusion. Immune exclusion involves SIgA preventing pathogens and antigens from gaining access to the intestinal epithelium. This suggests the decreased ability of SIgA to protect the mice from RV infection.

The presence of SFB in healthy (32) and diseased (64) adults indicates these microbes may have persisted from childhood. Based on the abundance of SFB relative to the absence of or incidence of disease, perhaps an appropriate and critical number of SFB may confer beneficial effects, and beyond this threshold, there might be detrimental effects. Whether the effects of SFB are specific to an age group or whether they continue to influence the GIT immune system later in life are unclear and remain as points of interest in the interaction of SFB in health and disease.

### Impact of Early-Life Nutrition on Ileal SFB Abundance

The timing of SFB abundance change at weaning (18, 25, 32) suggests that the inclusion of complementary foods may alter their abundance as well as their effects on the host epithelial and immune cells in the ileum. The absorptive function of the small intestine matures during weaning in infants (97) and seems to coincide with these changes in SFB (and other microbes) abundance in the ileum. SFB are proposed to obtain nutrients from the ileal lumen (12, 14, 21, 39, 55–57), and directly from the host. SFB may have evolved to attach to the ileal mucosa and derive nutrients in a cross-feeding manner with other bacteria residing in the inner mucus layer of the chemical barrier.

The abundance of SFB might increase with the increasingly diverse diet that infants consume from weaning, which might have subsequent effects on the immunological barrier maturation. However, the risks that an increase in SFB abundance may pose cannot be ignored. As discussed in section Immune-Mediated Disease, these microbes may be associated with some diseases (64, 86–89). However, observations from healthy SFB-mono-associated mice (21, 52), as well as healthy humans (1 day-old to 72 years old), have shown that SFB do not predispose them to disease, which suggests that changes in the profile of SFB abundance can occur without adverse pathological effects. These reports hint at the necessity of an appropriate bacterial load of SFB to avoid possible activation of putative pathogenic genes that these microbes may harbor.

SFB are thought to influence the production of immune cells, and this process may exacerbate immune-mediated diseases and explain the association with disease. Immune-mediated diseases may also likely have a reverse effect and trigger opportunistic pathogenicity in SFB, though this is yet to be investigated. The effects of a transient increase in SFB abundance cannot be inferred from studies with SFB-mono-colonized mice. The cause of the SFB abundance decrease post-weaning is still unknown. One suggestion is that the abundance of other anaerobic microbiota may rapidly increase and compete with SFB resulting in decreased SFB abundance. If SFB are indeed involved in immune barrier maturation, the decrease in SFB abundance may be due to the completion of immune barrier maturation which coincides with increased diet diversity (98–100). However, this still remains unclear. SFB also coexist with other microbes in the community and attempts to alter the abundance, even for a limited amount of time, may impact other members. It is therefore unclear how these potential interactions might affect the immune system and overall health.

### SFB Genes and Substrates

The analysis of the SFB genomes is necessary to infer the functions of predicted gene products and thereby model the utilization of substrates by SFB. Compared with selected *Clostridia*, annotation of SFB genomes indicated the absence of gene products involved in amino acid synthesis. Alternatively, the presence of genes coding for amino acid transporters and permeases implies a requirement for essential amino acids (4, 40, 41). *In silico* analyses of adult mouse and rat fecal SFB genomes revealed the presence of enzymes predicted to be involved in the glycolytic and pentose phosphate pathways. For the pentose phosphate pathway, enzymes for the oxidative phase were not predicted, though at least two catalases and one peroxiredoxin were detected which may contribute to the tolerance of SFB to the microaerobic environment of the small intestinal lumen (40). The predicted presence of these glycolytic and pentose phosphate enzymes suggests that carbohydrate macromolecules may be transported into the cell and utilized by SFB as an energy source.

Additionally, only a small fraction of enzymes required for synthesizing amino acids and co-factors have been predicted (40, 41). The lack of certain metabolic genes in the SFB genome and evidence that SFB reside in the nutrient-rich environment of the ileum may be indicative of ongoing reductive genome evolution and explain the reduced genome size (~1.57 Mb) compared to closely related *Clostrida* (3.97 Mb). Results from the published rat and mouse SFB genomes revealed the presence of carbohydrate, amino acid, protein, vitamin and mineral permeases, import/export transporters and ABC-type transporters (40). Continuous evolution within the SFB genome may have contributed to their commensalism as they are presently thought to acquire essential nutrients directly from the host.

The published rat and mouse SFB genomes (40) revealed predicted genes which reflect the transport and metabolism of carbohydrates. These include mannose, a component of the glycolytic pathway which is employed in the cell wall structure and malate–a source of carbon and ribose–used in cellular respiration. SFB are also thought to uptake cellobiose, ascorbate and fructose whose metabolism results in glyceraldehyde-3-phosphate of the glycolytic pathway. Moreover, the preferred niche of SFB is the ileum, where the brush border is located (40). This microvilli-covered surface on the ileal epithelium contains enzymes which degrade disaccharides into simple sugars that are then absorbed into the bloodstream (101). The presence of simple sugars in this region along with permeases and import/export-type transporters for sugars detected in the SFB genome points toward SFB being able to thrive on simple sugars (40).

An analysis of the SFB rat genome also indicated the presence of an N-acetylglucosaminidase family protein. N-acetylglucosaminidases are essential enzymes involved in the hydrolysis of complex oligosaccharides and their presence in the genome could point toward the ability of SFB to metabolize these kinds of carbohydrates. Analysis of the draft genome of human SFB from ileostomy patients, revealed the presence of one tentative extracellular N-acetylglucosaminidase, nine glycoside hydrolases representing six different families, as well as several cell surface-bound and extracellular proteases (102). Analysis of the human SFB draft genome indicated a lack of tricarboxylic acid cycle enzymes, similar to rat and mouse SFB. There were also no proteins identified that could be assumed to take part in the electron transport chain, confirming a fermentative lifestyle. An interesting observation was that human SFB contains genes for biotin synthesis (*bioA, B, D, F, W*, and *X*), which are lacking in rat and mouse SFB. This observation may demonstrate physiological or dietary differences of the hosts, or host microbial community, as some gut microbes can synthesize biotin while others cannot.

**Figure 4:**
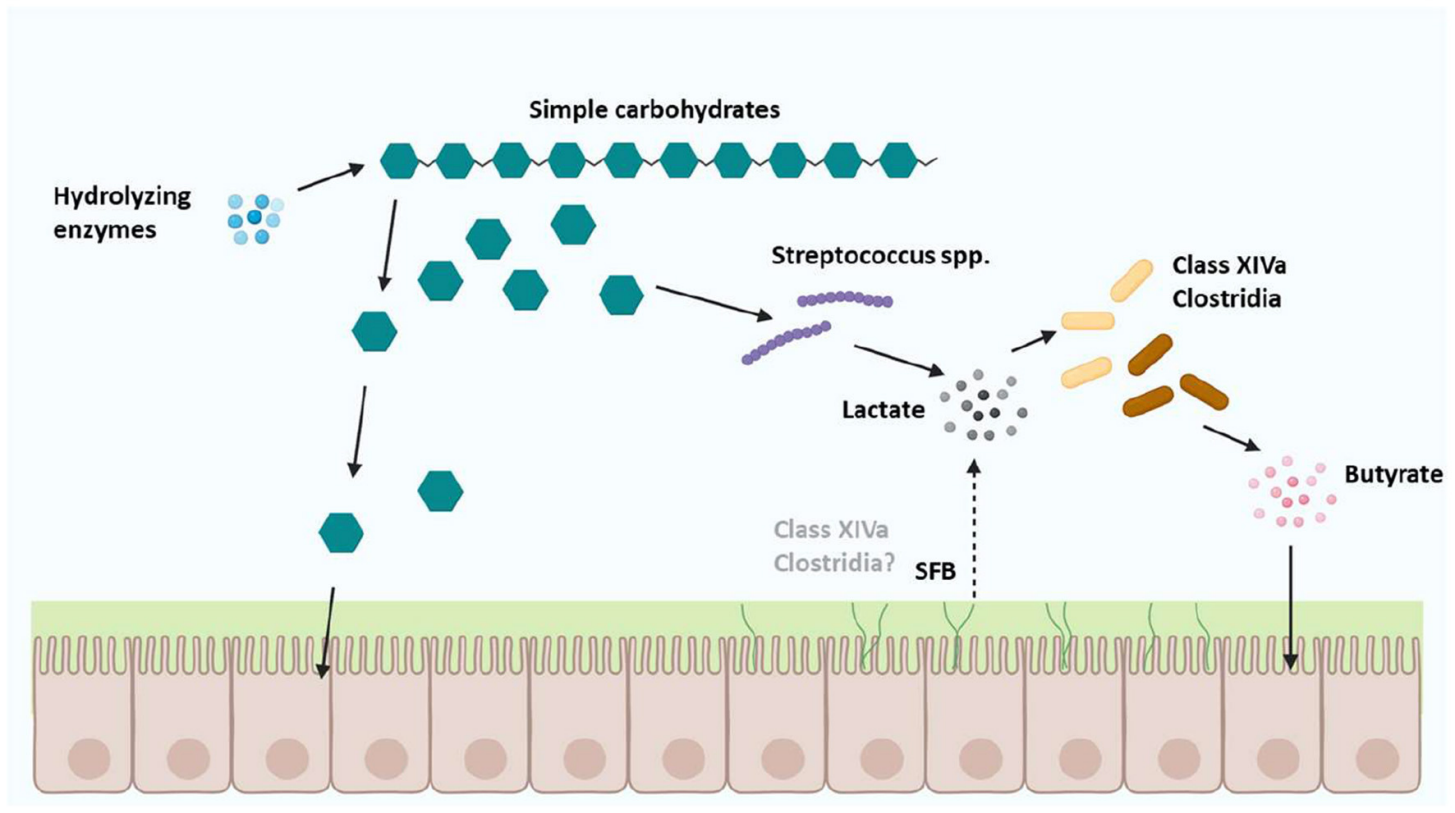
Cross-feeding of carbohydrate substrates between Clostridium class XIVa microbes and other Firmicutes. Cross-feeding with microbes from Clostridium class XIVa, and the Firmicutes phylum (42) might include SFB which are proposed to be a separate genus of Clostridia, based on a small number of SFB orthologs identified in host-associated Clostridia [Figure adapted with permission from Zoetendal et al. (4); Created with BioRender.com].

Zoetendal et al. (4) reasoned that the microbes from the *Clostridium* class XIVa, could utilize simple carbohydrate fermentation products from anaerobes such as some *Streptococcus spp* in a cross-feeding manner to produce butyrate (Figure 4). However, SFB reportedly metabolize pyruvate to produce acetate or ethanol but not butyrate evidenced by the lack of uptake transporters, though they reportedly metabolize pyruvate into lactate (40) like some *Streptococcus spp* (4).

Although diets post-weaning can contain low levels of simple sugars, whole-milk-based diets pre-weaning contain dairy carbohydrates such as lactose and oligosaccharides, but these diets did not increase ileal SFB abundance in mice at weaning, indicating that these metabolic pathways probably don't play a dominant role in SFB (39). Earlier work showed that mice fed a purified whole milk powder diet had lower SFB abundance in the ileum than those on a balanced composite diet. The composite diet comprised skim milk powder, Lucerne (alfalfa) meal, native corn starch, soybean oil, ground barley, fish meal, soybean protein concentrate, wheat middling's, corn protein concentrate, molasses, vitamin premix, mineral premix, calcium carbonate, and sodium chloride. These results suggest that the whole milk powder diet lacked additional nutrient(s) essential for optimum SFB growth. It is also possible that the reduced abundance of SFB in the mice receiving purified whole milk powder may have been an indirect effect, whereby some other microbe on which SFB rely are the primary effect (39).

Enriching a weaning diet would involve using a carbohydrate substrate which is typically added to the infant diet such as inulin. Inulin belongs to a class of soluble dietary fibers known as fructans and occurs naturally as a reserve carbohydrate in plants (103). It is digested by probiotics and encourages the growth of short-chain fatty acid (SCFA) producing microbiota (104, 105). Its unique chemical structure is made up of compounds with low chemical reactivity and resistant to digestion by the human GIT (106). Inulin is routinely added to infant formulas (107) mainly manufactured from bovine milk which, compared to human breastmilk (108), has a lower concentration of milk oligosaccharides (109). The complex molecular structures of these milk oligosaccharides are digested by probiotic bacteria which is key for developing a diverse and balanced microbial community in the infant GIT (110).

Inulin has also been reported to have several health benefits. These include reduced incidences of flatulence and bloating (111) and enhanced abundance of beneficial bacteria. Inulin promotes the production of SCFAs (acetate, propionate and butyrate) which creates an acidic environment that prevents the growth of pathogenic bacteria. SCFAs are also thought to be involved in immune system activation (112). Inulin has also been reported to improve epithelial integrity and barrier function and increase the expression of TJs (claudin-2 and occludin) (113). The reported production of acetate by SFB via the glycolytic pathway which is then converted to ethanol suggests that SFB may contribute some modulatory effects through SCFA production.

As an infant matures, a weaning diet is provided to complement breastmilk or formula to keep up with the nutritional requirements during development. The recommended weaning diet includes protein, fats, minerals, vitamins, and carbohydrate prebiotics, including inulin (114). Inulin has not previously been reported in SFB studies and can be easily incorporated in the diet in a dose-dependent manner to investigate the effects on SFB abundance. A mixture of inulin and fructooligosaccharides was tested in GF rats colonized by human fecal bacteria. The rats exhibited an increase in numbers of bacterial members from *Clostridium* class XIVa in the colon (105) but the effects of inulin specifically on SFB has not yet been investigated.

SFB are suggested to utilize nutrients in the ileum resulting from the consumption of a balanced diet, though the mechanism they use is still unclear. However, the reported presence of import and export transporters in the cell membrane of SFB filaments suggests this is how they uptake metabolic products (40). This process of nutrient import in SFB filaments may provide energy sources for the reported life cycle. The presence of orthologous flagellar genes in SFB implies similarities in bacterial flagellar morphology to Clostridium species and may include the arrangement of flagellin in the filament and attachment and nutrient uptake mechanisms (115).

### Future Perspectives

The difficulty in culturing SFB in the laboratory using the common microbiological techniques required alternatives to decipher the role of SFB in the ileum. SFB are unique as they require mucosal epithelial attachment to the ileum for survival. Their pattern of abundance change pre- and post-weaning in the ileum, as currently known, is also unique to them. Their preferred location of attachment in the ileum over Peyer's patches reiterates the importance of understanding the function of SFB. This niche of SFB in the ileum may position them to receive nutrients from digestion and influence the immune system. Whether SFB directly benefit the host GIT immune barrier or whether the host immune system triggers SFB to influence immunity in a feedback loop or enhance the action of other microbes remains unknown.

The reported influence of SFB on the immune system encouraged research on the effects of SFB presence or absence in immune-mediated disease. Perhaps in some cases, SFB contribute to the progression of the disease, though the reported absence of clostridial virulence genes imply that SFB may indeed offer protection from disease. Thus, far the results from these studies are varied and inconclusive, reflecting the complex association between SFB and the immune system.

The lack of knowledge of the complex relationship between SFB and the immune system is an important factor in dietary intervention studies. The proposal to enrich diets with substrates to manipulate the abundance of SFB is feasible compared to delivering SFB as a supplement (36). Hypotheses surrounding diet and SFB have been proposed (39), though there is a lack of knowledge about which dietary substrates affect SFB abundance. A prime candidate is carbohydrates (4, 40). Regardless of the impact on SFB abundance around weaning, observing the effects of a diet enriched in carbohydrate on the transient change in SFB abundance in infancy may give some clues on the role of SFB in immune barrier maturation.

Further investigation could lead to identifying how SFB interact with other microbes in the ileum. One group has suggested exploring the targeted use of metagenomic alteration of the gut microbiome by *in situ* conjugation (MAGIC) (116). They propose modifying SFB by harnessing naturally occurring horizontal gene transfer activity using an *Escherichia coli* strain as a donor to deliver engineered DNA. They reported achieving transient expression of the engineered DNA in the microbiome. It is unknown, however, whether SFB are naturally competent. Knowledge from this and more work on SFB may contribute to deducing the mechanisms by which these commensals uptake, utilize nutrients, and survive in the ileum. Understanding the function of SFB in the ileum may give more insight into the interaction of microbes with nutrition on the immune system.

The scarce knowledge on SFB is unsurprising, considering most studies have focused mainly on the effects of SFB presence and absence on the GIT immune system. Therefore, this new avenue of utilizing diet to decipher any influence on SFB could increase the understanding of the suggested role of these unique commensals in small intestine immune barrier maturation.

## Author Contributions

LO, RA, NR, and WM contributed to the conception of the review. The draft manuscript was prepared by LO. EA and NR contributed to the genomics portion of the review. All authors contributed to the preparation of the manuscript.

## Funding

LO was supported by a PhD Fellowship from the Riddet Institute Centre of Research Excellence, funded by the Tertiary Education Commission.

## Conflict of Interest

The authors declare that the research was conducted in the absence of any commercial or financial relationships that could be construed as a potential conflict of interest.

## Publisher's Note

All claims expressed in this article are solely those of the authors and do not necessarily represent those of their affiliated organizations, or those of the publisher, the editors and the reviewers. Any product that may be evaluated in this article, or claim that may be made by its manufacturer, is not guaranteed or endorsed by the publisher.
